# Expression of trypsin modulating oostatic factor (TMOF) in an entomopathogenic fungus increases its virulence towards *Anopheles gambiae* and reduces fecundity in the target mosquito

**DOI:** 10.1186/1756-3305-6-22

**Published:** 2013-01-21

**Authors:** Layla Kamareddine, Yanhua Fan, Mike A Osta, Nemat O Keyhani

**Affiliations:** 1 Department of Biology, American University of Beirut, Bliss Street, Beirut, 11072020, Lebanon; 2 Department of Microbiology and Cell Science, University of Florida, Gainesville, FL, 32611, USA

**Keywords:** Trypsin modulating oostatic factor, Biological control, *Beauveria bassiana*, *Anopheles gambiae*

## Abstract

**Background:**

Adult and larval mosquitoes regulate food digestion in their gut with trypsin modulating oostatic factor (TMOF), a decapeptide hormone synthesized by the ovaries and the neuroendocrine system. TMOF is currently being developed as a mosquitocide, however, delivery of the peptide to the mosquito remains a significant challenge. Entomopathogenic fungi offer a means for targeting mosquitoes with TMOF.

**Findings:**

The efficacy of wild type and transgenic *Beauveria bassiana* strains expressing *Aedes aegypti* TMOF (*Bb*-Aa1) were evaluated against larvae and sugar- and blood-fed adult *Anopheles gambiae* mosquitoes using insect bioassays. *Bb-*Aa1 displayed increased virulence against larvae, and sugar and blood fed adult *A. gambiae* when compared to the wild type parent strain. Median lethal dose (LD_50_) values decreased by ~20% for larvae, and ~40% for both sugar and blood-fed mosquitoes using *Bb*-Aa1 relative to the wild type parent. Median lethal time (LT_50_) values were lower for blood-fed compared to sugar-fed mosquitoes in infections with both wild type and *Bb*-Aa1. However, infection using *Bb*-Aa1 resulted in 15% to 25% reduction in LT_50_ values for sugar- and blood fed mosquitoes, and ~27% for larvae, respectively, relative to the wild type parent. In addition, infection with *Bb*-Aa1 resulted in a dramatic reduction in fecundity of the target mosquitoes.

**Conclusions:**

*B. bassiana* expressing *Ae. aegypti* TMOF exhibited increased virulence against *A. gambiae* compared to the wild type strain. These data expand the range and utility of entomopathogenic fungi expressing mosquito-specific molecules to improve their biological control activities against mosquito vectors of disease.

## Findings

### Background

Mosquito vectors transmit numerous diseases to humans and animals, causing illness and death that result in huge socio-economical burdens, especially in endemic countries. Control of mosquito vector populations has been almost exclusively based on the use of insecticidal chemicals; however, the strong dependence on insecticides for mosquito control worldwide and the use of such chemicals in agriculture has led to the physiological resistance of important mosquito vectors in recent years [1-3].

Entomopathogenic fungi, such as *Metarhizium anisopliae* and *Beauveria bassiana*, both EPA approved biological control agents (http://www.epa.gov/pesticides/), offer an environmentally friendly alternative to chemical insecticides, are virulent to mosquitoes, and have been considered as possible candidates for reducing disease transmission by insect vectors [4-6]. Despite their potential, widespread use of these fungi remains limited, due to a number of factors that include slow killing speed, low resistance to abiotic stress, issues of spore viability, and the amount of material (spores) needed for effective control. Nevertheless, there is still significant interest in using these biological control agents due to their effectiveness against insecticide resistant mosquito species [7] and advances in the development of tools and lures for their delivery to mosquitoes [8].

Several attempts at increasing the insecticidal effectiveness of entomopathogenic fungi have been based on the development of recombinant DNA techniques. In this context, transgenic entomopathogenic fungi were developed that express potent insect-specific neurotoxins that kill the infected host [9], or enzymes that enhance fungal resistance to adverse conditions such as UV [10], ultimately increasing spore viability in nature. In addition to increasing their insecticidal potential, entomopathogenic fungal strains can also be manipulated to target the pathogen, e.g. *Plasmodium* itself within its mosquito vector [11].

Recently, the idea of using targeted host-specific molecules to subvert normal insect development has been proposed [12,13]. Insects synthesize hormones and neuropeptides that have been exploited as targets for pest control. Examples of these include diuretic hormones and trypsin-modulating oostatic factors (TMOFs) that are found in insects including mosquitoes and flies. Administration of diuretic hormone increased secretion of fluids from the malphigian tubules leading to water loss and death of the insect. The *Ae. aegypti* TMOF (*Aea-*TMOF) was shown to circulate in the hemolymph, bind to gut receptors and inhibit trypsin biosynthesis by exerting a translational control on trypsin mRNA [14]. TMOF is resistant to proteolysis in the gut and easily traverses the gut epithelial cells into the hemolymph in adults and larvae, hence, it can be fed to mosquitoes resulting in inhibition of food digestion, anorexia, and is ultimately lethal to the insect [15-18]. In this report, we measured the virulence of a *B. bassiana* strain expressing *Aea*-TMOF to adult (sugar and blood-fed) and larval stages of *Anopheles gambiae*, the major malaria vector in Africa. The effect of this strain on mosquito fecundity was also determined.

### Methods

*Anopheles gambiae* G3 strain was reared as previously described [19]. Mosquito eggs were treated routinely with 1% Virkon for 4–5 minutes before floating them, to avoid spread of opportunistic infections in the colony. Wild type and *Aea*-TMOF expressing (*Bb*-Aa1) *B. bassiana* strains [20] were cultured at room temperature on potato dextrose agar (PDA) plates for a period of three to four weeks to allow growth of aerial conidia (spores). To collect spores, the surfaces of two *B. bassiana* PDA plates were scraped using a sterile cell scraper in the presence of sterile ddH_2_O and the extract was filtered over a glass wool packed column. The flow through material containing spores was centrifuged at 4500 rpm for 10 min and the pellet was washed twice with ddH_2_O. The pellet was resuspended in ddH_2_O containing 0.05% Tween 80, spores were counted with a Neubauer hemocytometer and adjusted to the appropriate concentrations for mosquito infections.

Adult mosquito bioassays were performed by spraying batches of 45 females (2-days old) each, with spore suspensions of *Bb*-Aa1 or the wild type (control) strain and mortality was scored on a daily basis. Briefly, adult mosquitoes were anaesthetized on ice, transferred onto a Whatman paper in a Petri dish, sprayed with the appropriate spore suspension using glass atomizers and then returned back to paper cups. Larval bioassays were conducted in plastic trays (8.5 × 13 × 5.5cm) each containing 40 three-days old larvae in a total of 110 ml water. Larvae were infected by applying different concentrations of conidia (1.1 × 10^9^, 5.5 × 10^8^, 2 × 10^8^ or 1 × 10^8^ conidia in a 50 μl olive oil suspension) to the surface of the assay chamber mixed to achieve homogenous surface spreading. The Kaplan-Meier survival test was used to calculate the percent mortality over the indicated time scale. Statistical significance of the observed differences was calculated by the Log-Rank test. Differences were considered to be significant if *P* < 0.05. Median lethal time (LT_50_) values for both *B. bassiana* strains were calculated from survival curves of adult mosquitoes (both sugar- and blood fed) and larvae infected with 1 × 10^8^ and 5 × 10^6^ spores/ml, respectively, using regression analysis. Statistical analysis of LT_50_ values was performed using the Student’s T-test. Median lethal dose (LD_50_) values were determined using concentrations ranging from 1 × 10^7^ to 2 × 10^8^ spores/ml in adult mosquitoes and 9 × 10^5^ to 1 × 10^7^ spores/ml in larvae using the Probit analysis. All experiments were repeated at least three times using different batches of mosquitoes and spores.

To examine the effect of fungal infection on mosquito fecundity, *A. gambiae* females sprayed with a suspension of 1 × 10^8^ spores/ml of wild type or *Bb*-Aa1 strain, were given a blood meal 24 h after fungal infection. Blood fed females were placed individually into paper cups and eggs were counted 48 h after blood feeding. Statistical analysis was performed using the Mann Whitney test.

### Results and discussion

**Figure 1:**
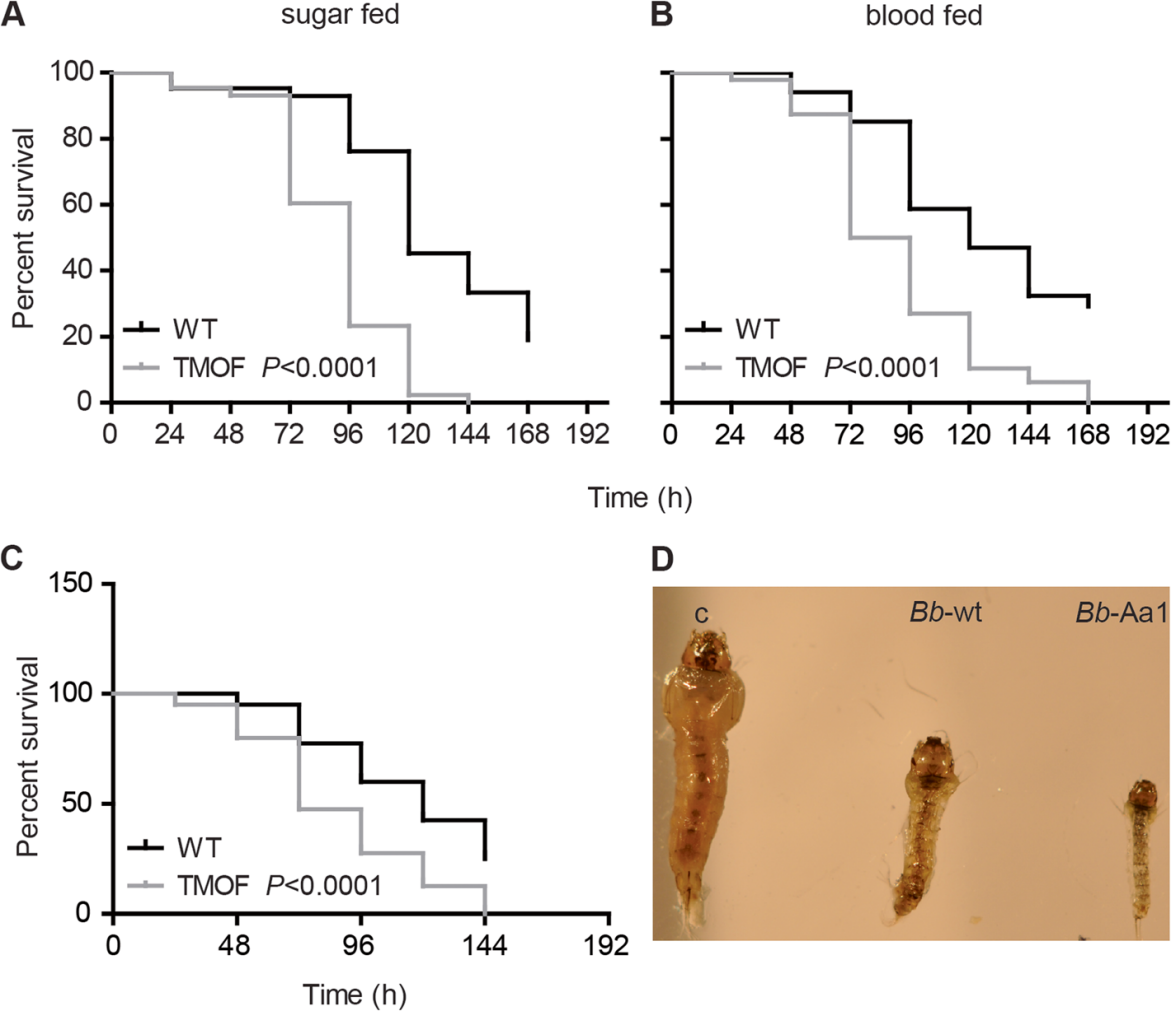
**Expression of *Aea*-TMOF increases *Beauveria bassiana *virulence to *Anopheles gambiae *mosquitoes. **(**A**) Sugar fed and (**B**) blood fed adult female *A. gambiae* mosquitoes were challenged with *Bb*-Aa1 strain expressing *Aea*-TMOF or the wild type parent, by spraying with a suspension of 1x10^8^ spores/ml and their survival was scored daily over the indicated period. (**C**) Three day old larvae were challenged by applying 5.5 x 10^8^ spores (suspended in 50 μl oil) to the water surface and their survival was scored daily over the indicated period as described in the Methods section. Graphs represent percent survival as calculated by the Kaplan-Meier method for one representative experiment of each group. Statistical significance was calculated by the log rank test and survival curves were considered significantly different if *P*<0.05. (**D**) Six days after *B. bassiana* infection, larvae showed significant growth retardation compared to non-infected controls. Wild type *B. bassiana* (*Bb*-wt); TMOF-expressing strain (*Bb*-Aa1). Data shown are representative of at least three independent biological replicates using different batches of mosquitoes and conidia.

*B. bassiana* strain *Bb-Aa1* expresses *Aea-*TMOF as a fusion protein with a 28 amino acid signal peptide derived from the *B. bassiana* chitinase gene to drive the extracellular secretion of the hormone [20]. Sugar and blood-fed adult, female *A. gambiae* as well as larvae were exposed to spores (conidia) of strain *Bb*-Aa1 or the wild type parent to determine the effect of *Aea*-TMOF expression on virulence. *Bb-Aa1* was more potent than its wild type parent against both sugar and blood-fed adults (Figure 1A and 1B), causing 40% reduction in LD_50_ values (50% mortality) in both groups compared to the wild type control (Table 1). However, LD_50_ values were similar between sugar and blood-fed mosquitoes infected with the same fungal strain, regardless of its type (*Bb*-Aa1 or wild type). Infection with *Bb*-Aa1 also induced a 15 and 25% reduction in the mean survival times (LT_50_ values) of sugar and blood-fed mosquitoes, respectively, compared to the wild type strain. LT_50_ values were also lower for blood- compared to sugar fed mosquitoes infected with the same strain, regardless its type. The fact that infections with the same strain, irrespective of its type, resulted in similar LD_50_ values between sugar and blood-fed mosquitoes, but lowered consistently the LT_50_ values for the blood- compared to sugar fed group, suggests that the blood meal itself does not seem to affect the virulence of a particular *B. bassiana* strain but rather mosquito tolerance to infection. Our data are in contrast to Myone *et al.* ([21]), who reported that blood-fed mosquitoes tended to be similar to or displayed greater median survival times than their unfed cohorts. There are several possible explanations for this discrepancy that can include the assay conditions (Myone *et al.* used 2 x 10^10^ conidia/m^2^ formulated in an oil suspension and sprayed onto sheets) and strain variation (whether fungal or mosquito), and this issue likely deserves greater attention.

**Table 1:** **LD_50_ and LT_50_ values of wild type and *Aea*-TMOF expressing *Bb*-Aa1 strain against *Anopheles gambiae***

**LD_50_ and LT_50_ values of wild-type and TMOF expressing *B. bassiana *infections in *Anopheles gambiae***			
**Strain**	**Mosquito stage**	**LD_50_ (conidia/ml)**	**LT_50_ (h)**
Wild-type *B. bassiana* (sf)	Adult	18.34 ± 2.86 x 10^7a^	133.26 ± 12.79^b^
Bb: spAeaTMOF (sf)	Adult	11.08 ± 2.79 x 10^7a^ (*P*=0.01)	113.23 ± 17.56^b^ (*P*=0.009)
Wild-type *B. bassiana* (bf)	Adult	18.00 ± 2.01 x 10^7c^	115.04 ± 11.26^d^
Bb: spAeaTMOF (bf)	Adult	11.31 ± 0.01 x 10^7c^ (*P*= 0.04)	86.48 ± 4.68^d^ (*P*=0.01)
Wild-type *B. bassiana*	Larvae	14.07 ± 1.24 x 10^7e^	116.31 ± 13.69^f^
Bb: spAeaTMOF	Larvae	11.32 ± 0.77 x 10^7e^ (*P*=0.01*)*	85.05 ± 14.66^f^ (*P*=0.001)

^a,c^ LD_50_ calculated from the 76 h time point.

^e^ LD_50_ calculated from the 48 h time point using the Probit analysis.

^b,d^ Bioassays performed by spraying mosquitoes with a concentration of 1x10^8^ spores/ml.

^f^ Larval bioassays performed using a concentration of 5x10^6^ spores/ml.

Statistical analysis was performed used the Student’s T-test and values were considered significant if *P*<0.05.

*A. gambiae* larvae infected with *Bb*-Aa1 exhibited greater compromised growth and survival as compared to those infected with the wild type strain (Figure 1C). *Bb*-Aa1 infection of larvae resulted in 20 and 27% reductions in LD_50_ and LT_50_ values, respectively, compared to the wild type strain (Table 1). Six days after *B. bassiana* infection, larvae exhibited significant growth retardation evidenced by their abnormally small size compared to non-infected controls; however, the phenotype was more severe in *Bb*-Aa1 infected larvae compared to those infected with the wild type strain (Figure 1D).

**Figure 2:**
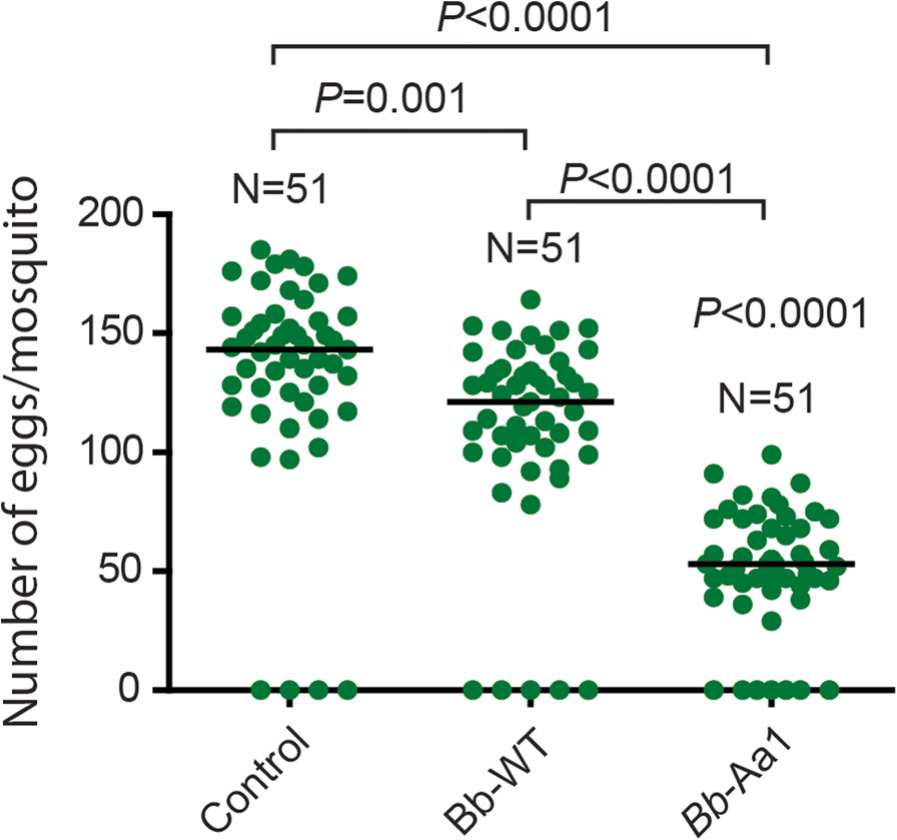
***Aea*-TMOF reduces fecundity of *Anopheles gambiae *mosquitoes. ***A. gambiae* females sprayed with a suspension of 1 × 10^8^ spores/ml of wild type or *Bb*-Aa1 strain, were given a blood meal 24 h after fungal infection. Non-infected, blood-fed mosquitoes were used as controls. Each circle on the graph represents the number of eggs per mosquito. Medians are indicated by black lines and distributions were compared using the Kolmogorov-Smirnov test. N, number of mosquitoes in each group.

Egg laying was significantly affected by fungal infection. Infection of *A. gambiae* mosquitoes with the wild-type *B. bassiana* strain resulted in a significant reduction (~16%) in fecundity compared to non-infected controls (Figure 2). However, expression of *Aea-*TMOF resulted in a dramatic reduction (~60%) in fecundity compared to controls (Figure 2). These data suggest that strain *Bb*-Aa1 also has the potential to reduce the size of *A. gambiae* mosquito populations by severely compromising fecundity.

*Aea*-TMOF does not induce vertebrate toxicity [22] and has passed EPA approval (http://www.epa.gov/pesticides/). Laboratory bioassays showed that the hormone is effective against all the major species of mosquitoes including anopheline [17]. Data from this as well as a previous study [20] indicate that expressing *Aea-*TMOF in entomopathogenic fungi is a promising approach for the delivery of the hormone to mosquitoes. Entomopathogenic fungi strongly affect mosquito survival [4,23,24] and may constitute a reliable measure for mosquito control when used as part of an integrated vector management plan. A field trial in a malaria endemic village in Tanzania, using experimental huts that simulate local houses, revealed that certain applications of fungal-treated surfaces lead to the infection of approximately 70% of mosquitoes entering the hut, which according to their model, is expected to reduce malaria transmission by 75-80% [25]. Of particular relevance to the data presented here, their model predicts that maintaining a high reduction in malaria transmission rates at lower levels of infected mosquitoes necessitates an increase in fungal virulence [25]. Hence, strains like *Bb*-Aa1 with improved virulence over standard parent strains are expected to strongly reduce malaria transmission even when the prevalence of fungus-infected mosquitoes is moderate.

Fungal strains with a narrower host range and coupled to specific TMOF peptides can also be constructed to maximize specificity and minimize non-target effects. TMOF is a physiologically important host hormone that must be expressed during the life-stages of adults and larvae to control digestion and growth [26], thus, the mechanisms for resistance to occur can be expected to be life threatening to the mosquito. Despite the fact that TMOF expression increases *B. bassiana* virulence to *A. gambiae*, LT_50_ values of *Bb*-Aa1 infected mosquitoes suggest that death is not quick enough to completely inhibit females from going through their gonotrophic cycles, even if spores were captured a couple of days before a blood feed. It has been proposed that insecticides exhibiting slow death rates that favor some reproductive success in female mosquitoes can suppress or at least reduce the emergence of resistance [5]. Further, since fungal pathogenesis is a multi-factorial process, where TMOF is not required, but acts to augment virulence, the likelihood of resistance is decreased due to the range of host processes targeted by the fungus, which would have to be overcome. Finally, expression of TMOF does not appear to increase the general virulence of *B. bassiana* to other insects, i.e. Lepidoptera, indicating target specificity [20]. Ideally, TMOF expressing fungi should be incorporated as part of integrated pest management (IPM) programs that do not rely on a single approach for insect control but which utilize compatible and synergistic approaches.

### Conclusions

Our data show that expression of (*Aea*)-TMOF in a mycoinsecticide can increase its effectiveness against two important mosquito vectors, *Ae. aegypti* and *A. gambiae*. Both survival times and the median lethal dose were lower in the Aea-TMOF expressing strain as compared to the wild type parent. In addition, target fecundity was dramatically reduced. Additional research is needed to examine issues of persistence, delivery, and even greater targeting of hosts, however, the principle of exploiting critical target host molecules for expression in an insect pathogen, which, during infection would compromise the host, can be expanded to a wide range of applications in the biological control of insects.

## Competing interests

The authors declare that they have no competing interests.

## Authors’ contributions

MAO and NOK conceived the study. YF constructed the fungal strains, LK performed the experimental work. All authors were involved in data analysis and interpretation. MAO and NOK drafted the manuscript. All authors read and approved the final version.
